# The Poor Man's Home.—VI

**Published:** 1897-09-25

**Authors:** 


### Modern Sociology.

### THE POOR MAN'S HOME.—VI.

### Health Laws in Many Lands (*continued*).

In France sanitary law dates from 1850, and the Act contains as its chief provision that in every community where the municipal council shall have declared it necessary at a special meeting, there will be named a commission to ascertain and report on unhealthy dwellings, and state the measures necessary for their improvement. The mayor is *ex officio* president of this commission, and its members must include a physician, an architect, and a member of the public relief bureau. The number of members varies from five upwards. In Paris it reaches 30. One-third of the commission resigns every two years; but as the members may be re-elected this does not necessarily alter the permanent nature of the commission. It is the duty of the commission to visit places which are reported to be unhealthy, and suggest the remedies required. Owners, or persons otherwise interested, are allowed a month in which to protest against the condemnation of the property. Unfortunately, the appointment of the sanitary commission is optional, and very few municipal councils have taken advantage of it. Indeed, only eight out of the 36,000 administrative sub-divisions of France have so far established one. The reason is sufficiently obvious. The initiative rests with the municipal council; and as Dr. Gould, the American health commissioner, says, "The elect of universal suffrage are not over ready to take part in any movement which may bring them in conflict with the interests of their influential constituents. Furthermore, the posts being non-remunerative, there is difficulty in finding men with sufficient time on their hands, or men far enough removed from extraneous influences, who are competent to undertake the duties assigned them."

Moreover, the Act itself is very incomplete. It contains no provision for the improvement of offices, stores, and such places, where people pass their days but do not sleep. No summary procedure is possible. It takes from six months to a year to dispose even of cases in which no opposition is offered, and where landlords protest a remedy may be indefinitely delayed. Even when the protest is disallowed the power to coerce or punish the proprietor is limited, nor has the commune powers to effect improvements themselves. To exemplify the dilatoriness and futility of the law one case may be quoted. On August 24th, 1886, a complaint was laid before the Prefecture of the Seine that a certain dwelling was damp. Twenty-two different communications followed in the various steps of carrying out the law. The last bore the date of June 2nd, 1891 and stated that the proprietor had been fined 25 francs. Some critics declare that the Act of 1850 has rather hindered the improvement of dwellings. Certainly it cannot have helped to any extent.

In Belgium the state of affairs is much the same as in France. There is, indeed, a superior council of hygiene and a medical commission for each province, but as neither has any executive power they can do little. The burgomaster, in towns of over 2,000 inhabitants, has power to issue a closing order in the case of unhealthy houses. As a matter of fact, however, the burgomaster is rarely a sanitary reformer, and has not too keen an eye and nose for insanitary dwellings.

In Germany sanitary administration, like most other matters, lies in the hands of the police. There is, indeed, a municipal commission, and in the larger cities a sanitary commission, but these can only advise. Nor can a sanitary commission be formed except on the initiative of the chief of police. The powers of the police are, however, great, and sharply exercised. The direct responsibility of keeping property clean and healthy is laid on the landlord. If he finds his tenants keep their houses in a filthy state, he must warn them to clean them. If they do not do so, he must turn them out. If he fails to do this the sanitary authority will, if neighbours complain, clean the premises for him, and charge him with the cost. Although rather tyrannical, this process is better for public health than the dilatory methods in vogue in France.

It would be impossible to make any general statements as to sanitary laws in America. No one code prevails over the whole Western Continent. But the law of New York may be quoted as being the most thorough and complete in America, if not in the world. The department of health is divided into two bureaus. One deals merely with vital statistics; but at the head of the other is the sanitary superintendent, who must be a medical man of ten years' standing. The board over which he presides consists of four members, and it takes a majority of three to order a house to be vacated. But as a matter of fact the board is almost always unanimous. Under the sanitary superintendent's control is a force of forty inspectors, half of whom are doctors and half experts in various departments of sanitation. In addition to these, forty-five policemen, who have had not less than five years' service, are detailed for work under the board of health. These are employed specially to see that the provisions of the health law are properly carried out in tenements and lodging-houses.

One of the chief provisions of the law is that which insists that there shall be at least six hundred cubic feet of air to each occupant of a room. Where less than this is afforded the house is regarded as over-crowded. It is also stipulated that in every room or set of apartments occupied by one family there shall be either an open fire-place or a place for a stove properly connected with a chimney. The ceilings of rooms are to be not less than eight feet above the floor. The regulations with regard to windows insist that the window or windows shall equal in extent one-tenth of the superficial area of the room, and that the sash shall open to the extent of twelve square feet. It seems, however, that by special permission of the board of health, the admission of light and air from another room or hall will be tolerated. This seems a dangerous concession. There are very stringent regulations as to the supply of water on each floor, and as to the proper use and condition of ash-pits and sanitary conveniences. To secure ventilation it is insisted that between dwellings of one storey high there shall be a clear space of 10 feet; this space is to be increased to 15 when the building is two storeys, to 20 when it rises to three storeys, and to 25 when it is higher. Considering what summer heat means in New York the allowance is scanty enough.

